# Alignment of Lyapunov Vectors: A Quantitative Criterion to Predict Catastrophes?

**DOI:** 10.1038/srep37102

**Published:** 2016-11-15

**Authors:** Marcus W. Beims, Jason A. C. Gallas

**Affiliations:** 1 Departamento de Física, Universidade Federal do Paraná, 81531-990 Curitiba, Brazil; 2 Departamento de Física, Universidade Federal da Paraíba, 58051-970 João Pessoa, Brazil; 3 Instituto de Altos Estudos da Paraíba, Rua Silvino Lopes 419-2502, 58039-190 João Pessoa, Brazil; 4 Max Planck Institute for the Physics of Complex Systems, Nöthnitzer Str. 38, 01187 Dresden, Germany; 5 Institute for Multiscale Simulation, Friedrich-Alexander-Universität Erlangen-Nürnberg, 91052 Erlangen, Germany

## Abstract

We argue that the alignment of Lyapunov vectors provides a quantitative criterion to predict *catastrophes*, i.e. the imminence of large-amplitude events in chaotic time-series of observables generated by sets of ordinary differential equations. Explicit predictions are reported for a Rössler oscillator and for a semiconductor laser with optoelectronic feedback.

The prediction of catastrophic events is arguably one of the most important open problems in physics and in the natural sciences. Catastrophes can be roughly defined as short-duration, large-amplitude events which follow and are followed by relatively long and random intervals of *maturation*^1^,^2^. The great challenge is, of course, to anticipate the duration of the random quiescent intervals and thus to predict impinging undesirable events such as earthquakes, tsunamis, rogue waves, market crashes, political crisis, etc. Considerable work was done in the last years to understand the mechanisms behind the emergence of large-amplitude pulses^3^. An idea of the intense activity concerning *outliers* in several distinct lasers, in nanophotonic devices and media, in excitable systems, and in other key applications may be obtained by perusing a small selection of representative papers published in the last two years^4^,^5^,^6^,^7^,^8^,^9^,^10^,^11^,^12^,^13^,^14^,^15^.

Our aim here is to introduce a *quantitative indicator* capable of anticipating the imminence of large-amplitude events in chaotic time series. As far as we know, there exists hitherto no quantitative criterion for predicting such large-amplitude events. As defined below, the quantitative measure proposed here is based on the so-called Lyapunov vectors (LVs)^16^,^17^,^18^,^19^,^20^,^21^, namely on useful vectors which provide a step-by-step big-data record of what exactly happens with the angles between stable and unstable manifolds during the whole evolution of a given dynamical process. As shown below explicitly, we find the onset of large peaks in a physical variable of interest to be related to the *alignment of LVs along the flow direction*. Therefore, since Lyapunov vector alignment precedes large peaks, it can be used to predict the later. Our findings are empirical observations based on numerical computations and, therefore, the conclusions presented here are simply conjectures.

Lyapunov vectors are attracting growing interest nowadays and providing new insight about the dynamics of complex systems. For instance, LVs deliver not only the familiar spectrum of Lyapunov exponents, but also angles between stable and unstable invariant manifolds for every point along the temporal evolution. LVs are obtained by integrating the equations of motion forward and backward in time along the same trajectory according to algorithms described in the literature^16^,^17^,^18^,^19^,^20^,^21^. So far, LVs have been fruitfully applied to rather distinct situations, from turbulence governed by partial differential equations to the evolution of interacting neutrino gas^16^,^22^,^23^,^24^, from properties of thermal reservoirs to cluster synchronization in scale-free networks^21^,^25^, from traffic of vehicles to ocean-atmosphere modeling^18^,^26^, and from self-similarity and angle-enhanced bifurcation diagrams to dissipative holes in conservative tori^20^,^27^.

Before proceeding, we mention that much work has been done recently concerning phenomena called *extreme events* independently of whether they are governed by partial differential equations or not. In works related to extreme events and in particular in the field of laser dynamics, there is no consensus on what would be a good definition of an extreme event. Some refer to a definition taken from hydrodynamics and consider as extreme a threshold for the amplitude of a pulse at twice the abnormality index. Others consider that large-amplitude pulse means that the amplitude is larger than the mean-amplitude of the pulses plus a few values of the standard deviation. And there are also papers where one looks to the deviation of the statistics of the pulse amplitude as a parameter varies. For a review, see the collection of review papers presented by Akhmediev *et al.*^3^. Here, however, we are not concerned with extreme events but, instead, with large-amplitude pulses commonly observed in experiments and analysed using small sets of ordinary differential equations.

## Results

As described in *Methods*, the angles between LVs are calculated from the expression^20^


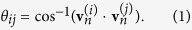


Our illustrative examples below involve chaotic dynamics and three Lyapunov exponents 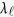 ordered such that *λ*_1_ > 0, *λ*_2_ = 0 and *λ*_3_ < 0. One has the following situations for the angles between LVs: (i) *θ*_13_ is the angle between the unstable and stable manifolds, (ii) *θ*_23_ is the angle between the stable manifold and the flow direction, (iii) *θ*_12_ is the angle between the unstable manifold and the flow direction.

### Catastrophes in Rössler’s oscillator

While studying flows in three dimensions, Rössler was interested in reinjection mechanism where a trajectory, after having a slow motion in one part of a manifold in the phase space, jumps through large excursions to another branch of the manifold. A prototype set of equations with the minimum ingredients for producing sequences of such large excursions is^28^:


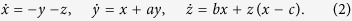


Here, (*x*, *y*, *z*) are real variables evolving continuously as a function of time, (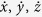) are the corresponding velocities, and (*a*, *b*, *c*) are real parameters controlling the oscillator. These equations give rise to oscillations of *x* and *y* which are amplified for *a* > 0 and result in a spiraling-out motion. Such outbound oscillations are coupled to the *z* variable in a nonlinear way which induces the reinjection back to the center region, with the cycle of spiraling-out and reinjection repeating indefinitely.

**Figure 1:**
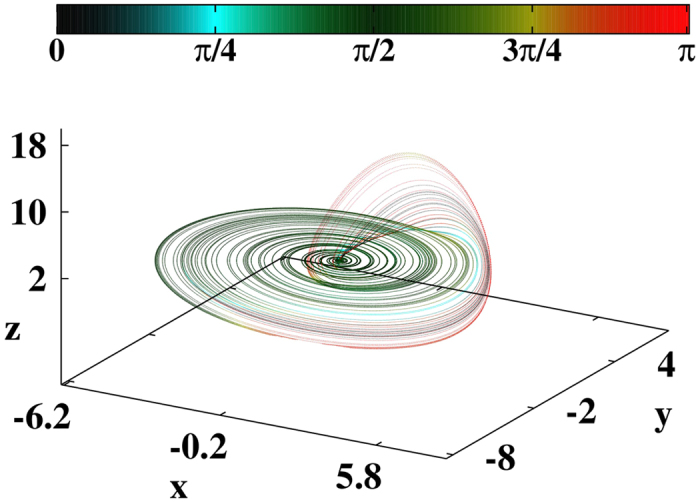


Figure 1 displays a typical chaotic trajectory, obtained for *a* = 0.38, *b* = 0.3, and *c* = 4.82 and initial condition near the unstable fixed point *x* = *y* = *z* = 0. Colors represent the values of the aforementioned angle *θ*_23_. Parallel to the *xy* plane there is spiraling-out motion, represented in green (*θ*_23_ ~ *π*/2), while the reinjection loops are shown in black and red, associated with *θ*_23_ approaching either 0 or *π*, respectively. The attractor in Fig. 1 was obtained solving Eq. (2) numerically with the standard fourth-order Runge-Kutta algorithm and fixed time-step *h* = 0.05. The first 10^7^ steps were discarded as transient, with LVs computed subsequently for 8 × 10^5^ steps (including forward and backward time motion). The chaotic trajectory shown is of *screw type*^28^ with irregular oscillations in amplitude and in reinjection times.

The time-series of the representative trajectory shown in Fig. 1 consists of large pulses along the *z*-axis (associated with a homoclinic orbit^30^) and are separated by irregular time intervals during which the trajectory has chaotic oscillations basically confined to the (*x*, *y*) plane. The large peaks in the *z*-axis start just before the trajectory gets reinjected back near the origin (i.e. near to the unstable fixed point *x* = *y* = *z* = 0). Our purpose is to show that using the angle between the LVs it is possible to predict when large peaks in the *z*-axis occur. From Fig. 1 one sees clearly that 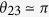 well before the large peaks emerge out of the (*x*, *y*) plane.

**Figure 2:**
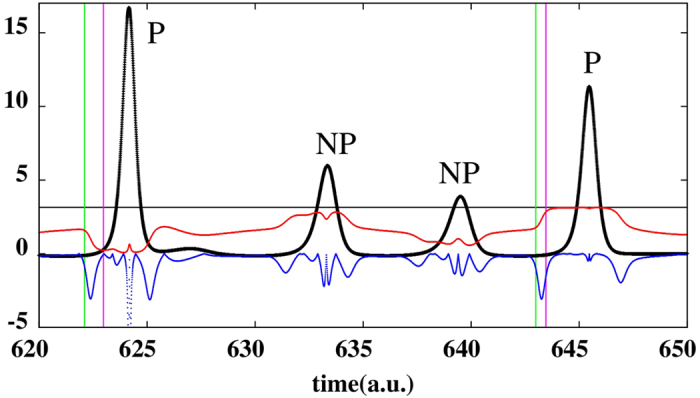
Temporal evolution of *z* (black line) for Rössler’s oscillator. The red trace shows *θ*_23_ while the blue trace depicts 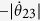. P refers to *predicted* as large spike while NP refers to a peak detected but *non-predicted* as large, according to the choice of thresholds (see text). The horizontal line marks *π*.

Figure 2 illustrates the relation between the LVs and the emergence of large spikes. The black curve shows the temporal evolution of *z*, the red line displays *θ*_23_, and the blue line depicts the variation of the derivative 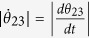. For easier visualization, we plot 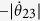. In this range, *z* presents two large peaks (with *z* > 10, marked P) and two intermediate peaks (with *z* ~ 5, marked NP). The blue curve shows that for all four peaks the quantity 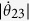 has a local minimum slightly before the peaks. The beginning of two such minima is indicated by vertical green lines. The key here is to observe that before large peaks in *z*, fast variations of 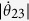 occur when, in the red curve, *θ*_23_ rapidly changes either from *π*/2 → 0 (leftmost peak) or from *π*/2 → *π* (rightmost peak).

The above observations suggest the interplay of two conditions prior to the occurrence of spikes. First, an *alert condition* occurs when the presence of a minimum in 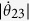 signals to an imminent spike but without indication of its amplitude. A pair of alert conditions are indicated by the vertical green lines in Fig. 2 (using 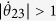). Second, an effective *catastrophe condition* is detected by inspecting how close *θ*_23_ approaches either 0 or *π*, defined by two suitable *thresholds* 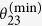 and 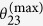. This catastrophe condition corresponds to LV *alignment condition*, i.e. when the stable manifold aligns along the direction of flow thereby providing information concerning the intensity of the imminent spike. The detection of a pair of catastrophes is indicated by vertical magenta lines in Fig. 2 (using 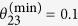 and 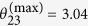). Accordingly, peaks labeled P (predicted catastrophes) satisfy both alert and catastrophe conditions, while peaks labeled NP (non-predicted catastrophes) satisfy only the alert condition. Of course, the discrimination between P and NP can be controlled by tuning the aforementioned thresholds.

**Figure 3:**
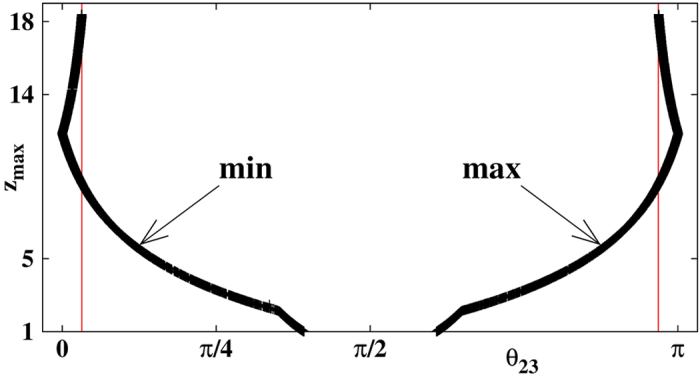
Maximum of *z* spikes as a function of *θ*_23_. The vertical lines mark 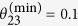 and 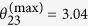 discussed in the text. Although the sequence of *z*_max_ is randomly distributed in time, both branches are symmetric.

To quantify the relation between the LV alignment condition and the intensity of the spikes, Fig. 3 displays local maxima *z* > 1 as a function of the *θ*_23_ maxima (close to *π*) and minima (close to 0) for Rössler’s oscillator (Fig. 2). The two vertical red lines mark the thresholds 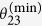 and 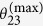. To obtain Fig. 3 we evolved the trajectory for a long time checking when maxima (minima) of *θ*_23_ were larger (smaller) than 1.9 (1.2). After this, we computed the maximum (minimum) of *θ*_23_ until a peak in *z* appeared. In other words, we determined how close *θ*_23_ approaches 0 (or *π*) near peaks with *z* > 1. From Fig. 3 one sees that all peaks with large *z* values are associated with angles *θ*_23_ close to either 0 or *π*.

To assess the performance of the alignment conditions over extended intervals of time the trajectory was integrated much longer, for *t* = 5 × 10^4^ time steps. The LV alignment was checked automatically using a pair of threshold values 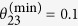 and 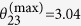. In this way, a total of 3745 peaks were found to obey the alert condition 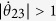, i.e. peaks which are potentially catastrophes. Of this total, 2372 where indeed found to be P (predicted) catastrophes, namely *z*_max_ > 9.11, the value corresponding to the crossing in Fig. 3 of the red lines with the min and max curves. The remaining 1373 spikes were smaller NP (non predicted) events. Spikes in the classes P and NP were separated by imposing numerically the alarm and LV alignment conditions. Thus, *all detected spikes were correctly accounted for*. In addition, on average we determined alert times to precede spikes by ~2.2 time units, and catastrophe times to precede by ~0.85 time units. Of course, much earlier prediction times are obtained using less stringent values for 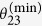 and 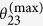. For instance, using 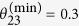 and 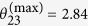 the catastrophe prediction time increases to 1.51, almost doubles, on the average. In this case 2944 peaks of type P were detected. We remark that the large interval of integration chosen above was used simply to check that the method was able to detect the imminence of large pulses to get a feeling for the distribution of large events in the model considered. Large integration intervals are by no means needed to predict catastrophes.

### Catastrophes in semiconductor laser with optoelectronic feedback

Trajectories for Rössler’s equations are known to be closely related to the presence of Shilnikov homoclinic orbits in the system^28^,^29^,^30^. To check a possible influence of homoclinicity on the prediction times, in the next example we consider a situation where Shilnikov’s theorem does not apply^31^,^32^, namely we consider a semiconductor laser with optoelectronic feedback. The aim here is to show that the alignment of LVs is able to predict large peaks also in absence of homoclinicity. In dimensionless units the laser is governed by the equations^31^,^32^


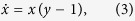



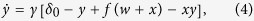



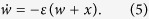


Here, the variables *x*, *y*, *w* are proportional to the photon density, the carrier density, and the laser intensity, respectively, while 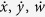 are corresponding rate of change. The feedback is controlled by *f* = *α(w* + *x*)/(1 + *s(w* + *x*)), *γ* is the ratio between the population relaxation rate and the photon detuning, *ε* is the high-pass frequency in the feedback loop, *δ*_0_ is the solitary laser threshold, *s* is a saturation coefficient of the amplifier and *α* is a coefficient proportional to the photodetector responsivity. In our simulations we fix *α* = 1, *γ* = 1 × 10^−3^, *ε* = 2 × 10^−5^, *δ*_0_ = 1.017 and *s* = 11, values for which chaotic dynamics is observed experimentally^32^.

As before, Eqs (3)–(5), 26, 27 were solved numerically with the fourth-order Runge-Kutta algorithm and fixed time-step *h* = 0.05. The first 10^7^ time-steps were discarded as transient and the LVs were computed for 9.5 × 10^5^ times-steps (including forward and backward time motion).

**Figure 4:**
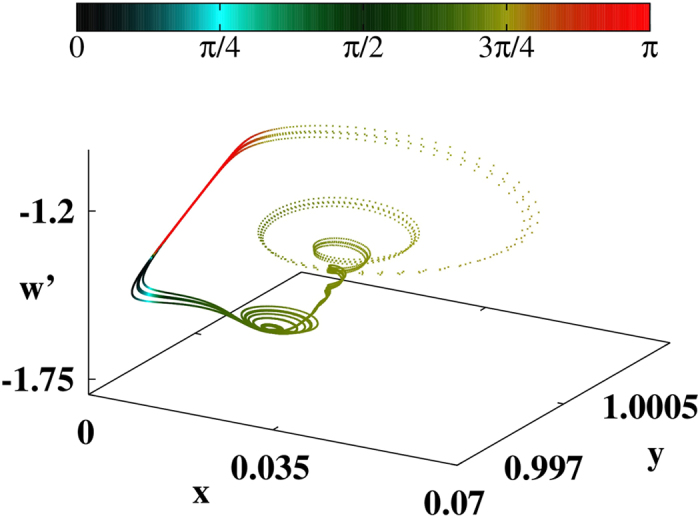
Phase-space evolution of the chaotic semiconductor laser attractor with colors indicating *θ*_23_. See text.

**Figure 5:**
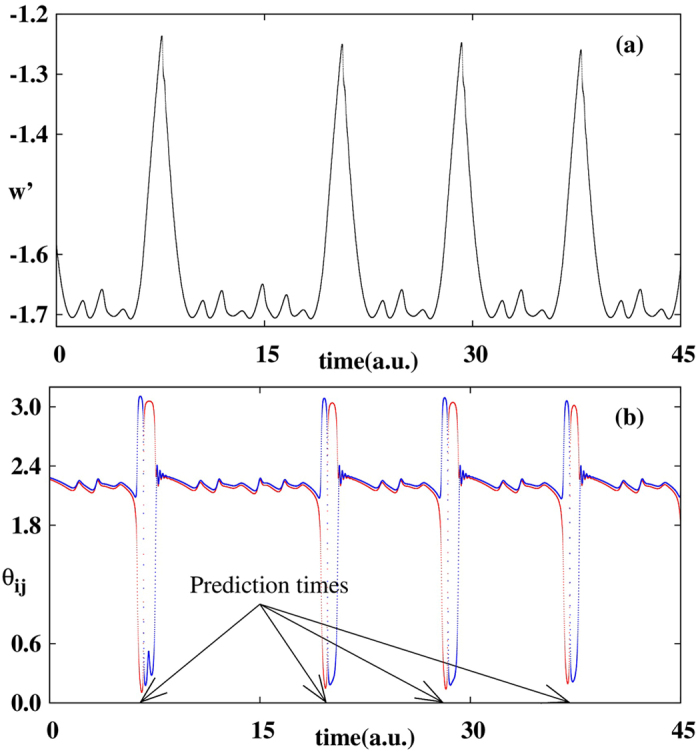
Temporal evolution of **(a)** large laser pulses **(b)** *θ*_12_ (red trace) and *θ*_23_ (blue trace). LVs correctly anticipate large laser spikes.

Figure 4 shows a typical trajectory with colors representing the values of *θ*_23_ for every point of the trajectory. We started from (*x*_0_, *y*_0_, *w*_0_) = (1, 1.5, 0.5) and observed a small spiraling-out motion in the (*x*, *y*) coordinates, then an abrupt increase of *w*, and then a larger spiraling-in motion leading to the reinjection process. It is easy to realize that when *w* increases, *θ*_23_ changes colors and assumes values approaching 0 or *π*. This can be more easily observed in the time evolution of *w* shown in Fig. 5 (in units of 10^4^ time intervals). Figure 5(a) displays the scaled variable *w*′ = *w* × 100 and Fig. 5(b), for the same times, presents the *θ*_12_ (red line) and *θ*_23_ (blue line). Comparing Fig. 5(a) and (b) we observe that preceding all large peaks in *w*, one finds *θ*_12_ → 0 and, simultaneously, *θ*_23_ → *π*. Note that in contrast to the Rössler’s system, in this case both stable and unstable manifolds align along the direction of flow. Thus, once again, the LVs from the stable and unstable manifolds tend to align along the flow direction before large-amplitude events. In other words, large peaks of *w* occur shortly *after* the alignment of stable and unstable manifold along the flow direction. As seen in Fig. 5(b), in this case both the alert and catastrophe times (i.e. prediction times) can be close to each other. On average, prediction of large peaks occurs at about 1 time unit (10^4^ time-steps).

## Discussion

The alignment of LVs along the flow direction provides a straightforward and apparently reliable means of predicting catastrophic events in chaotic dynamical systems. This was corroborated for two familiar dynamical systems, namely a Rössler oscillator, and a semiconductor laser with electronic feedback. We are not aware of any other quantitative criterion able to predict large-amplitude events.

To predict large-amplitude events, one should evolve a trajectory and monitor 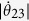. Whenever its magnitude becomes larger than a suitable system-dependent threshold, a peak should be expected. To estimate the peak intensity, evolve the trajectory further and check how close *θ*_23_ approaches 0 or *π*. The closeness of the approach defines the peak intensity and, therefore, can anticipate a catastrophe. In realistic situations where noise is present, the validity of the alignment conditions depends on the survival of the spikes under the noise influence.

An open challenge is to check the effectiveness of LVs alignment to predict catastrophes for higher-dimensional systems, a considerably more complicated framework for which there are yet no efficient methods to compute angles between invariant manifolds. Nevertheless, from a theoretical point of view we anticipate no problems in predicting catastrophes regardless of the underlying dimensionality of the system. This, of course, remains to be explored.

## Methods

Here, we describe briefly the essential details for the numerical implementation of the procedure to compute the angle between the two invariant subspaces of a 3D dissipative dynamical system. We are interested in the forward evolution in the tangent space of an initially orthonormal basis 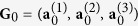. Such evolution is governed by the equation


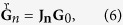


where **J**_*n*_ = **J**_*n*−1_**J**_*n*−2_ … **J**_0_ is the product of the Jacobian matrix of the map evaluated for every orbital point. It is implicit here that at the initial time *n* = 0 we have already computed the orbit for a sufficiently long time (forward transient time) so that the orthonormal basis of the tangent space converged already to the asymptotic Gram-Schmidt (GS) vectors^17^. In order to avoid divergences the matrix 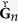 is renormalized for each step *n*. As usual, this can be done by the QR decomposition


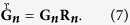


The matrix **R**_*n*_ is upper-triangular


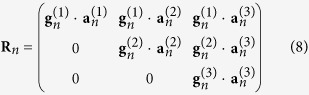


and contains the information obtained in the GS orthonormalization procedure of 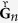. Here, 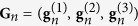 are the GS vectors after the orthonormalization. Since 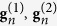 and 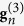 are orthonormal by construction, they can only provide information about the local rates of expansion (contraction) of the vectors, stored in the diagonal elements of **R**_*n*_, from which the standard Lyapunov exponents can be obtained. However, to calculate the angle between the invariant subspaces in the tangent space, we need the LVs determined from the relations


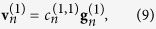



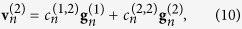



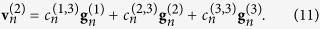


The coefficients 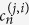 are determined from the dynamics in tangent space for the backward direction. In the matrix form we have 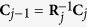, where 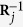 is the inverse of the **R**_*j*_ matrix obtained from the GS orthonormalization procedure in the forward evolution, Eq. (8). The time *j* starts to count after the backward transient, which is the time in the backward evolution sufficiently long to converge the tangent initial conditions close to the LVs. The LVs have normalized length so that the columns of 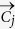 must be normalized to 1. The initial condition for **C**_*n*_, before starting the backward evolution, can be a generic nonsingular upper triangular matrix, which is the GS basis.

## Additional Information

**How to cite this article**: Beims, M. W. and Gallas, J. A. C. Alignment of Lyapunov Vectors: A Quantitative Criterion to Predict Catastrophes? *Sci. Rep.* **6**, 37102; doi: 10.1038/srep37102 (2016).

**Publisher’s note:** Springer Nature remains neutral with regard to jurisdictional claims in published maps and institutional affiliations.
